# “You kind of want to fix it don’t you?” Exploring general practice trainees’ experiences of managing patients with medically unexplained symptoms

**DOI:** 10.1186/s12909-015-0523-y

**Published:** 2016-01-25

**Authors:** Mary Howman, Kate Walters, Joe Rosenthal, Rola Ajjawi, Marta Buszewicz

**Affiliations:** Department of Primary Care and Population Health, UCL (Royal Free Campus), Upper Third Floor, Rowland Hill Street, London, NW32PF UK; Research Department of Primary Care and Population Health, UCL (Royal Free Campus), Upper Third Floor, Rowland Hill Street, London, NW32PF UK; Centre for Medical Education, Dundee Medical School, The Mackenzie Building, Kirsty Semple Way, Dundee, DD2 4BF UK

**Keywords:** Medically unexplained symptoms, Somatisation, Medical Education, General practice, Mixed methods research

## Abstract

**Background:**

Much of a General Practitioner’s (GP) workload consists of managing patients with medically unexplained symptoms (MUS). GP trainees are often taking responsibility for looking after people with MUS for the first time and so are well placed to reflect on this and the preparation they have had for it; their views have not been documented in detail in the literature. This study aimed to explore GP trainees’ clinical and educational experiences of managing people presenting with MUS.

**Method:**

A mixed methods approach was adopted. All trainees from four London GP vocational training schemes were invited to take part in a questionnaire and in-depth semi-structured interviews. The questionnaire explored educational and clinical experiences and attitudes towards MUS using Likert scales and free text responses. The interviews explored the origins of these views and experiences in more detail and documented ideas about optimising training about MUS. Interviews were analysed using the framework analysis approach.

**Results:**

Eighty questionnaires out of 120 (67 %) were returned and a purposive sample of 15 trainees interviewed. Results suggested most trainees struggled to manage the uncertainty inherent in MUS consultations, feeling they often over-investigated or referred for their own reassurance. They described difficulty in broaching possible psychological aspects and/or providing appropriate explanations to patients for their symptoms. They thought that more preparation was needed throughout their training. Some had more positive experiences and found such consultations rewarding, usually after several consultations and developing a relationship with the patient.

**Conclusion:**

Managing MUS is a common problem for GP trainees and results in a disproportionate amount of anxiety, frustration and uncertainty. Their training needs to better reflect their clinical experience to prepare them for managing such scenarios, which should also improve patient care.

**Electronic supplementary material:**

The online version of this article (doi:10.1186/s12909-015-0523-y) contains supplementary material, which is available to authorized users.

## Background

People present frequently to both primary and secondary care with medically unexplained symptoms [1, 2]. MUS describes symptoms with no clear organic cause. Although often considered a diagnosis of exclusion, this does not necessarily need to involve investigation and referral depending on the clinical history and examination and the possibility of MUS should be considered in all consultations where an organic diagnosis is uncertain. Such symptoms make up a large proportion of clinical workload involving between 15-39 % of consultations [3]. The term MUS includes chronic somatisation (multiple, recurrent and frequently changing physical symptoms usually present for several years defined by the ICD-10) and somatic symptom disorder as classfied by DSM 5 [4, 5].

There have been questionnaire surveys of practicing GPs on their attitudes to MUS in the UK, Spain and Pakistan [6–8]. Results suggest that GPs find MUS presentations difficult to manage, although they generally consider it the GP's role to do so by reassuring the patient, and acting as a gatekeeper to further investigation and referral.

Other studies have used in-depth interviews to explore consultations with patients with MUS in more detail [9, 10]. These studies described GPs’ frustration as they were unable to meet patient expectations and their concern that they would sometimes investigate or refer due to patient demand rather than clinical indication. They usually tried to offer explanations for patients’ symptoms, but around half felt unable to discuss possible underlying psychosocial factors and struggled with the time needed for these consultations.

Dowrick et al. illustrated how consultations around MUS can be dysfunctional, with GPs dismissing patients’ symptoms without explanation, or organising investigations or referrals with the aim of terminating the consultation [11]. Unnecessary investigations or referrals can result in adverse outcomes for patients and place a significant financial strain on health services [12].

Despite the pervasiveness of MUS, educational literature on teaching clinicians about this topic is relatively sparse. Surveys conducted in the US and UK indicate that undergraduate teaching about MUS is variable and, if present, often consists of a single lecture during a Psychiatry programme [13, 14]. A recent qualitative study in the UK found feelings of frustration and hopelessness towards MUS amongst medical students who often described little formal teaching on the topic, with learning through experience sometimes involving poor role-modelling from seniors [15].

Most postgraduate educational literature focuses on qualified GPs rather than trainees and finds no consistency in approach. Studies in the UK and Denmark have focussed on teaching the “reattribution” technique which aims to enable the patient to feel understood, broadening the agenda beyond physical symptoms, making the link with psychosocial issues and negotiating further treatment [16–23]. However there was no evidence of improved patient outcomes [21] and reattribution is only applicable in a minority of cases [24].

Smith et al. have also produced research based on a review of the literature suggesting managing patients presenting regularly with MUS with a multidimensional approach. This involves intensive assessments at the start lasting 60–90 minutes and can be performed by primary care physicians or nurse practitioners. It utilises a collaborative approach, development of the provider-patient relationship and cognitive behavioural techniques including realistic goal setting, clear explanation of symptoms and negotiation of a specific treatment plan [25]. A pilot study using four primary care physicians trained in these techniques suggested a positive impact on managing MUS [26]. A programme in the US focussed on training 63 first year primary care residents (first year postgraduates) in interview techniques and assessing psycho-social factors. Results showed these graduates were more confident in managing MUS but effects on patient satisfaction and outcome were too small to be considered meaningful. [27].

There has been no detailed qualitative work looking at the attitudes and experiences of GP trainees with regard to MUS. These trainees are at the interface between education and practice, often having overall responsibility for managing people with MUS for the first time. An in-depth understanding of GP trainees’ experiences and attitudes towards patients with MUS could help us develop tailored educational strategies.

Three research questions were addressed:

1. What are GP trainees’ attitudes and feelings towards managing patients with medically unexplained symptoms?
2. What management strategies do they use in managing people with MUS and what are their experiences of managing MUS?
3. What are GP trainees' perceptions of the teaching they have received on this topic, and how it could be improved?

## Methods

This was a mixed methods study involving both questionnaires and in-depth interviews, using the pragmatic paradigm [28]. This involves chosing data collection and analysis methods which are most likely to provide insight into the central research question [29]. An explanatory method was used with data from the questionnaires being analysed and then the interviews being used to further illuminate attitudes and experiences documented in the questionnaires [30].

### Ethics

Ethical approval was given by Camden and Islington REC, reference number 09/H0722/79. Principles of informed consent, non-coercion and right to withdraw were followed in compliance with the Helsinki Declaration.

### Participants

The study population consisted of 120 GP trainee attendees at an educational session about MUS from four London vocational training schemes (weekly educational meetings attended by doctors training to be GPs). They came from all three years of GP training, so some were specialty trainees in hospital posts in year 1 or 2 of GP training, while others were based in general practice in their third and final year of training.

### Phase 1 Questionnaire: data collection and analysis

Trainees were asked to complete a paper-based questionnaire examining their attitudes to MUS and their previous educational experience in this topic prior to receiving the training session. This attitudinal questionnaire was based on a questionnaire piloted and used by Rosendal et al. with 43 Danish GPs [22, 31, 32]. We considered this to be the most recent, user-friendly and attitude-focussed of similar questionnaires and consisted of 24 questions with responses on a visual analogue scale. In order to address the research questions on education some free text questions were added and the number of questions on attitudes reduced to 10 in order that the questionnaire did not take too long to complete (Additional file 1). The questionnaires were anonymised and values described and tabulated using mean and median scores. Free text comments were analysed thematically by the first author.

Following administration of the questionnaire a teaching session was delivered by two of the researchers (MH and MB) who had no other educational or supervisory relationship to the trainees. The educational session lasted 2.5 hours and used video and written case scenarios to promote discussion about the presentation and management of people with MUS. Other authors (KW, JR and RA) had no involvement in the teaching or contact with the trainees.

### Phase 2 Interviews: data collection and analysis

The same trainees were then approached by email eight weeks after receiving the educational session about MUS and invited to take part in in-depth qualitative interviews. Those who did not reply within two weeks were emailed once more. All those who consented were interviewed by the first author with reference to a topic guide which covered: their understanding, experiences and attitudes towards managing patients with MUS, examples of managing people with MUS in their clinical practice, their perceptions of good management and barriers to achieving this. Experiences of previous teaching about MUS and their views about how the topic should be taught were also explored. The majority of interviews were conducted at their place of work with a few conducted at the trainee’s home. They ranged from 26 to 61 minutes with an average of 40 minutes. Participants were offered a £20 book voucher to thank them for their time.

The interviews were audio-recorded, anonymised and transcribed verbatim. Data were analysed by thematic analysis using the framework approach [33]. This involved inductive coding of data by all the researchers, with four looking at all the transcribed data (MH, KW, JR, MB) and one a sample (RA). There was iterative discussion and negotiation of the coding framework with respect to the research questions, in order to identify key themes and subthemes from within the data [33]. The coding was sensitive to *what* was said and also *how* it was said (specifically the linguistic use of pronouns and metaphor), in exploring the interplay between these to describe how the trainees make sense of their experiences [34]. Analysis remained grounded in the data collected and included searches for disconfirming evidence. RA is a medical educationalist and the other four researchers are GPs.

## Results

### Phase 1 – questionnaire results

#### Characteristics of respondents

80/120 (67 %) of the trainees completed the baseline questionnaire. No information is available on non-respondents. The majority were female with 12 male respondants (15 %). Seventeen (21 %) were in their first year of GP training, 29 (36 %) in their second year and 28 (35 %) in their final year of training. Six were graduates (8 %).

The vast majority (76 %) of trainees completing the questionnaires said that they first started clearly identifying or managing patients with MUS after qualification, with only 8 % saying they had experience of this whilst undergraduates. Eight per cent stated that they were not aware of seeing or managing patients with MUS until ST3 level (i.e. 5 years after qualification). The majority of trainees (59 %) reported that they now saw people with MUS at least weekly, which is still much lower than the percentage of people identified as presenting to general practice with MUS in epidemiological studies [3].

#### Questionnaire responses

Table 1 shows the responses of the trainees to the attitudinal questionnaire. The responses were mixed and relatively neutral in tone, with a lack of strongly positive feelings towards MUS consultations in general, but also a relative lack of more strongly negative ones.

**Table 1:** Attitudes to patients with MUS 1 (Percentages to nearest 0.1)

Questionnaire stem	*n*	Percent^a^ with Likert scale response 1–3 (not at all, a little bit, a little)	Percent^a^ with Likert scale response 4–5 (somewhat, a fair amount)	Percent^a^ with Likert scale response 6–7 (much, very much)
I feel angry	79	83.5 (66/79)	15.2 (12/79)	1.3 (1/79)
I worry about missing illness and being sued	79	25.3 (20/79)	45.6 (36/79)	29.1 (23/79)
I often feel unsure of what to do	79	13.9 (11/79)	64.6 (51/79)	21.5 (17/79)
I feel comfortable	79	64.6 (51/79)	30.4 (24/79)	5.1 (4/79)
I enjoy working with them	79	68.4 (54/79)	31.6 (25/79)	0.0 (0/79)
I feel anxious	78	50.0 (39/78)	37.2 (29/78)	12.8 (10/78)
I am confident in my approach	79	55.7 (44/79)	39.2 (31/79)	5.1 (4/79)
I resent seeing them	79	81.0 (64/79)	15.2 (12/79)	3.8 (3/79)
I think they take up too much of my time	79	68.4 (54/79)	24.1 (19/79)	7.6 (6/79)
I sometimes use cognitive behavioural therapy techniques	77	71.4 (55/77)	26.0 (20/77)	2.6 (2/77)

^a^Percentages to nearest 0.1

Most trainees did not feel well prepared for managing people with MUS, with a mean score of 4.3 (+/− 1.8) on a Likert scale asking them how prepared they felt, with over a quarter (23/80, 28.8 %), reporting having no previous teaching on the topic. The majority (53/80, 66.3 %) said they had received some undergraduate teaching, with the majority (29/53, 54.7 %) noting that this was in the form of a lecture during mental health teaching. A few (4/53, 7.5 %) mentioned having some consultant case based discussion during medical school or teaching during communication skills. Individual students recalled receiving teaching about MUS during accident and emergency, general practice, neurology and palliative care and one during their BSc. Only 12/80 (15.0 %) said they had received any postgraduate teaching and this was usually discussion with their GP trainer or during a Psychiatry post.

The free text comments on the questionnaires also indicated that the trainees felt under-prepared for managing people with MUS. Several reiterated the lack of formal teaching while others noted difficulties in following up patients so they could find out whether they had organic pathology or not. Some examples are as follows:

*“Although we come across them in our training they are often labelled as heartsink and no real advice given as to how best help such patients.”*

*“(I am) unsure how far to take investigations, how to broach the subject that it may be psychological rather than physical.”*

### Phase 2 - Qualitative Interviews

Fifteen GP trainees participated in phase 2 of the study. Table 2 compares the characteristics of those interviewed with those completing the questionnaire. There was a higher percentage of ST3s (GP Registrars, working wholly in general practice) in those interviewed. Those interviewed had graduated from a total of 7 medical schools and attended 4 vocational training schemes (VTS).

**Table 2:** Characteristics of those interviewed compared to the whole sample completing questionnaire

Participant characteristic	Number of those completing questionnaire (80 in total)	Number of those agreeing to interview (15 in total)
Gender – men, *n* (%)	12 (15)	2 (13)
Year of GP training		
1,*n* (%)	17 (21)	2 (9)
2,*n* (%)	29 (36)	3 (20)
3,*n* (%)	28 (35)	10 (66)

We report the themes emerging from the interviews in three key areas: 1) Feelings engendered by patients with MUS 2) Management of patients with MUS 3) Education about MUS

#### 1. Feelings engendered by patients with MUS

Participants indicated that patients presenting with MUS made up a significant part of their workload. The majority of patients described were those with multiple symptoms presenting frequently and likely to have chronic problems. Trainees reported a range of feelings towards them, from negative to more positive, with uncertainty, fear of misdiagnosis and a sense of impotence identified as key explanations for the negative emotions experienced.

Most trainees described consultations with patients with MUS as challenging, often provoking emotions of anxiety, frustration, unease, feeling overwhelmed and sometimes anger.

*“I could find myself getting really agitated with him and getting cross as well … I’ve got lots of other things to do. I don’t have time for this.”* (ID 2)

### Uncertainty and fear of missing disease

Difficulty dealing with uncertainty appeared to underpin much of the unease described by trainees.

“*I guess for me that’s the whole uncertainty … letting people go out the door and … it could be a brain tumour but it’s probably just a stress headache … coping with that* (uncertainty) *is something that I do struggle with.*” (ID 4)

Interviewees frequently mentioned their concern that they might be missing a diagnosis, with some attributing this to inexperience. A few expressed concern about litigation.

*“I found that I would more doubt myself, that there’s something here that I’m missing. I can’t explain it, but that doesn’t mean it’s not explainable.”* (ID 1)

### Impotence and the need for action

Several participants described a sense of dissatisfaction or failure at their inability to make a diagnosis or alleviate a patient’s symptoms. In both examples below there is an interesting shift in the use of pronouns from ‘I’ to ‘we’ (the medical profession) as a stronger justification for not knowing the diagnosis or being able to ‘fix’ things.

*“I was thinking, God, he wants me to be able to diagnose what these sensations are caused by. We don’t know. He’s had the tests and they’re all normal.”* (ID 3)

*“The last thing you want to do is leave your patients in pain. It would be lovely if I could get rid of her pain, then all she had to deal with is the rest of her life. Realistically a large proportion of it we can’t actually fix.”* (ID 6)

The metaphor of the *body as a machine* that can be fixed was used several times, with trainees generally feeling much more comfortable when they were able to find a problem, fix it and see an improvement.

*“There is something quite nice about being able to say, ‘Oh you’ve got epigastric pain, yes you’re H pylori positive, I can do something about it.' You kind of want to fix it don’t you?”* (ID 13)

However such a drive to “fix” things, possibly initiated and perpetuated through current medical education, may be detrimental to trainees’ ability to be comfortable with patients with MUS and manage them effectively. For example, quite a few indicated that simple empathetic listening to the patient did not feel sufficiently therapeutic and appeared to want to offer more concrete action.

*“We feel that we’re not getting anywhere, and that there’s nothing I can actually do for her other than listen, and I’ve listened for 15–20 minutes.” (ID 6)*

### More positive attitudes to patients with MUS

However, a few of those interviewed felt quite positive about managing patients with MUS and had developed strategies to work with such patients, which they found effective.

“*It’s very satisfying when you have worked with patients like this … no-one has really bothered to work with them before … to gradually get people off being so focused on that particular symptom is really satisfying because you think 'I've seen them change.'”* (ID 3)

Those who appeared to cope better with managing patients with MUS seemed more able to operate outside the biomedical model and to have more realistic goals than fixing or curing the patient.

*“I think for me a positive outcome would be that the patient accepts their symptoms and accepts that maybe we don’t know what it is and we can’t do anything about it.”* (ID 3)

#### 2. Management of patients with MUS

The sophistication of management strategies described by participants varied. Three key themes were identified: emphasis on MUS as a diagnosis of exclusion, reticence to broach psychological issues and limited explanations given.

### Emphasis on MUS as a diagnosis of exclusion

Most of the participants interviewed appeared to view MUS as a diagnosis of exclusion. They wanted to be as clear as possible that they were not missing a diagnosis and struggled to tolerate uncertainty.

*“I think once I’ve reassured myself that it’s something that doesn’t require further investigation, that it’s not something very serious, then I … put on a different hat with a different lot of skills and manage that patient quite differently, but I would need that reassurance … to make sure I’m not missing something.”* (ID 14)

The trainees appeared to get reassurance from the absence of ‘red flags’ (symptoms/signs or patterns that might indicate a serious cause) in most cases. Whilst trainees described using the history and examination to exclude red flags, arranging a large number of investigations also appeared to be a way of dealing with their uncertainty. Some reflected that some investigations were likely to be initiated to allay the doctor’s anxiety rather than for the benefit of patients, causing feelings of conflict and unease in the doctor.

*“It’s a horrible balance investigating their symptoms when it’s not needed is reinforcing their anxieties, their concerns, when it’s irrelevant and just putting people under extra stress. It’s not just simple procedures sometimes … they can be fairly traumatic experiences for people. So I guess it’s thinking why am I doing this? Is it merely so I feel a bit more comfortable?”* (ID 2)

*“It’s a way of ending the consultation … to send her for bloods or a chest X-ray.”* (ID 4)

In other cases trainees described doing a test they felt sure would be negative in order to try to reassure the patient, sometimes advising the patient first that it was likely to be negative. Several trainees raised the possible pitfalls of investigations, including abnormal but irrelevant findings and perpetuating patient requests for tests.

*“You offer them a test … it’s normal, they then want something else because you’ve not really explored the idea that actually there isn’t anything organic and … it’s quite hard for them to move on past the, ‘there’s something wrong with me’, so they want another test to find out what it is. And I do think that’s not necessarily their fault. That’s probably our fault.”* (ID 1)

Many also described using referrals as a way of absolving themselves of uncertainty. In contrast to arranging investigations, trainees on the whole described referral as a positive strategy, despite reporting several examples of a patient being dissatisfied following referral.

*“Maybe it’s better to refer, have that one assessment and then you’re done rather than ten GP appointments.”* (ID 1)

Only one participant suggested that referral to secondary care might have negative consequences for the patient.

*“I think if I felt helpless and was still uncertain about whether this could be somatisation ..… but if you can’t get an appointment for three months … they’re sitting there getting more and more worried … thinking there’s something so wrong with them they have to go and see a specialist.”* (ID 11)

There were a few exceptions to the predominant strategy of initially excluding physical causes for the patient’s symptoms, with a couple of trainees describing an approach which focused on eliciting the patient’s concerns and any accompanying psychological factors from an early stage and using these to inform their management.

*“Really trying to pay attention to the psychological side of things early on … ask them about what else is going on in their life.”* (ID 4)

There was no clear pattern regarding stage of training or prior experience to explain why some trainees appeared to use the biopsychosocial model more than others in managing patients with MUS. Several described management becoming less problematic with experience and time spent with the patient.

*“I’d … end up making less referrals because I’d talk to her about why* (she wants a referral) *and actually there are often things that you can work through in primary care without having to actually refer them on.”* (ID 1)

### Reticence to broach psychological issues

Although most felt there was likely to be a psychological component underlying many presentations, many trainees were not keen to broach this with patients even after excluding red flags. Some seemed unclear about the role of a psychiatrist or psychologist in MUS while others assumed the patient would not engage with a conversation about psychological factors, and so did not raise it.

*“It all seemed very physical. And I think I hadn’t quite got to the end of investigations … I never raised it* (anxiety/depression) *with her and she’s never raised it with me. I don’t think it would have added anything.”* (ID 1)

Over half cited concerns about damaging the doctor/patient relationship if suggesting a referral for help with psychological difficulties.

*“They think they’re completely normal and they present with something physical and you say 'Well actually I’m going to send you to the Psychiatrist' … then that’s not going to be great for your relationship.”* (ID 11)

However, although some of the trainees felt that a lack of time meant deeper exploration of a patient’s psychosocial background was difficult, others were able to explore underlying psychological factors over several consultations.

*“And then we finally got to the root of this (presentation) … probably about six months after she first consulted … she came back and said the real problem is she has panic attacks … and there’s all of this which is … underneath … but it takes you a while getting there.”* (ID 9)

A few described feeling that seeing the patient regularly and giving them time to talk was useful in itself.

*“It’s actually quite therapeutic for her just to come in and complain.”* (ID 1)

### Limited Explanations given

There was a range in the complexity of explanations given to patients to account for their symptoms, with a suggestion that those trainees earlier in their training found it more difficult to give a satisfactory explanation than those with more GP experience. A few offered no explanation.

Most trainees described offering reassurance that the symptoms were likely due to a non-serious but unidentified cause.

“*It’s not medically explained and sometimes you get aches and pains that we can’t find a reason for but try not to worry about it.”* (ID 13)

Several described sharing their uncertainty with patients; whilst a few offered more complex explanations, for example describing how emotions may cause physical symptoms or linking poor sleep with physical symptoms.

*“Most people have a form of stress in their lives and … if you explore that they can sometimes see … that the stress from that makes them tense and then you can say that sometimes the tension in your muscles is what’s making you feel tired.”* (ID 6)

#### 3. Education about MUS

Trainees discussed the lack of preparation within both undergraduate and postgraduate curricula for dealing with patients with MUS. Much of their learning was through role modelling of other doctors. They generally considered that their medical training had prepared them for managing more concrete presentations, making diagnoses and being able to “fix” the patient.

*“In medical school you’re taught that patients have things wrong with them and there’s always a medical cause for it and you’ve got to try and find it.”* (ID 4)

Perhaps as a consequence of an emphasis on the medical model, some trainees described being surprised by the reality of clinical practice.

“*And suddenly when you step into the doctor’s role, I think in training you get, you assume that you can make those feelings go away because you can control stuff. And I don’t think you can really.”* (ID 12)

However, there was the occasional exception who recalled being taught about MUS having a very meaningful impact on them at medical school.

*“I remember it* (MUS teaching) *being a very interesting afternoon … immediately you could think of patients … and the feeling of being completely out of your depth, and it was someone actually addressing something I think we all get very nervous about. It was a big deal because it changed the way you … had these set ways of thinking.”* (ID 11)

Several felt they had learnt to over-investigate during their hospital jobs with mixed role-modelling of both positive and negative attitudes and management from consultants in the hospital.

*“One of my professors would* (say) *'we’ll do a CT anyway because at least then we’ve done everything and there’s nothing more to do.'”* (ID 8)

*“One of the consultants … was sympathetic, nice about the whole thing, 'you're here now with this pain and we'll deal with this pain now.' Whereas others have been a bit more sharp and short about the whole thing. 'You're here with this pain. We've done everything. Here's your painkillers.'* (ID 15)

## Discussion

### Summary of main findings

Our findings identify common feelings of frustration and anxiety in GP trainees when dealing with patients with MUS as a result of not being able to clearly ‘fix’ the patients’ problems and so feeling impotent about how to proceed. Management of patients with MUS by trainees often involves exhaustive investigation or referral to exclude all other potential organic and biomedical causes before either dismissing the patient with the ‘reassurance’ of normal tests, or in a few cases exploring potential psychosocial factors. This fragmentation of care seems to be communicated through medical school through the prioritising of scientific and biomedical origins of diagnoses and very limited focus on MUS and the biopsychosocial model [35]. Role modelling can also play an important role in this. The findings from interviews were similar to those from the questionnaire, although with more diverse and strongly expressed views, both positive and negative. This may reflect the fact that a greater proportion of those interviewed were in their final year of GP training and so had had longer to form their opinions and more experience to base these on, or that those with stronger opinions were more likely to agree to be interviewed.

### Comparison with the literature

Our findings mirror previous studies with qualified GPs, both in terms of finding such consultations challenging and also in the sub-optimal management of patients; for example describing particular difficulties in giving satisfactory explanations for their symptoms or exploring psychosocial aspects of their presentation [6, 9, 10, 36]. Several of our interviewees described consultations which fitted the model of dysfunctionality around MUS illustrated by Dowrick et al. with attempts made to “quickly reassure” patients, or doctors feeling pressured into ordering investigations in order to end the consultation [11]. GP trainees differed from qualified GPs in previous studies in more often assuming that they might miss diagnoses due to their lack of experience, possibly leading to a tendency to over-investigate and a keenness to refer for a second opinion. This reflects the finding that doctors often try to manage uncertainty with action rather than inaction, and memorable cases where a diagnosis was missed may affect subsequent decision making disproportionately [37].

There is no strong evidence base for how teaching about MUS should be conducted. Studies have generally focused on clinician or student evaluation rather than patient outcomes [38, 39]. Studies about teaching ‘reattribution techniques’ for patients with MUS were viewed positively by clinicians but not found to have a positive patient impact [36]. Smith et al.’s work on teaching primary care physicians and nurse practitioners to utilise techniques including CBT has suggested some postive impact on patient management in pilot studies in the US [26, 40]. Whether there is scope for these methods to be used in UK primary care would need further evaluation: one potential problem is the time needed for initial assessments and also the availablity of nurse practitioners to help with these. They also target the more frequent attenders with MUS so those at the more severe end of the MUS spectrum.

### Strengths and limitations

This is the first in-depth mixed methods study documenting the educational and clinical experiences of GP trainees about MUS. Use of two methods (questionnaire and in-depth interviews) helped strengthen the quality of the data obtained through triangulation.

The study was small and London-based so may not be generalisable to other geographical areas. Basing the data collection around a teaching session and having a GP tutor conduct the interviews may have influenced the results, with participants potentially wanting to impress the interviewer by repeating ideas expressed in the lecture. The study was conducted in this way in order to access the trainees and encourage participation. However, the questionnaire was anonymous, and the interviewer was aware of potential bias and deliberately probed for a range of perspectives.

There may have been selection bias in those who volunteered to be interviewed, possibly tending towards those with stronger attitudes to patients with MUS, either positive or negative. Most participants were women, although this proportion mirrored the gender proportion in the VTS groups. We selected from across a range of 4 VTS schemes and doctors had graduated from 7 medical schools.

### Implications of the current study

Uncertainty cannot be removed from the consultation and medical education needs to improve teaching around managing uncertainty and decision-making [37]. Bleakley calls for a new type of education integrating medical science with patient care to help make the transition from student to doctor [41]. He describes the importance of both practical knowledge and learning to help understand the patient. Educational methods focusing on this would surely help reduce the false separation of biomedical and psychosocial in students’ minds, so that rather than putting on “*different hats*” to address these they can offer a more integrated, holistic approach to the patient.

Structured formal education around MUS, managing uncertainty and reducing unnecessary investigations and referrals is likely to be helpful, starting in medical school by challenging notions that all symptoms can be medically explained and building on this as students gain more clinical experience. There is some teaching of the biopsychosocial model in the UK, particularly in Psychiatry, but perhaps this needs to be emphasised and extended across specialities. In the US reseachers have proposed a curriculum for medical postgraduate trainees that focuses on psychosocial assessment and providing good mental health care [42]. Similar emphasis is needed across specialties in the UK, both in undergraduate and postgraduate curricula.

The informal curriculum, encompassing skills learned by observation or practical experience, is likely to be equally important, with situational coaching around specific cases in the workplace helping students to calibrate their decision-making about specific patients. GP trainers and other medical educators need to recognise the important role they play in guiding their trainees to reason effectively and holistically regarding patient care. Within both undergraduate and postgraduate training an increased emphasis on psychosocial aspects of patients’ presentations, continuity of care and therapeutic listening is likely to be of use, as well as focusing on giving effective and empowering explanations rather than simple “reassurance” [43].

To be effective this is likely to require a range of teaching techniques within the formal and informal curriculum. Communication skills teaching was described as being particularly useful by our participants, as well as case discussion and experiential learning. As there are differences in attitudes to patients with MUS in different countries, for example with a greater proportion of doctors in Pakistan feeling they are missing a physical diagnosis in patients with MUS than in the UK, different teaching strategies are likely to be needed in different countries [8].

In part the terminology of MUS may be problematic, as often clinicians take it to be a diagnosis of exclusion by investigation. It could be argued that labelling the presentation as MUS promotes compartmentalisation of the patient’s narrative, but equally labelling may be needed in order to promote teaching of it as a concept. Only 59 % of trainees in the questionnaire reported seeing patients with MUS at least weekly which is low compared to epidemiological studies. This may reflect a lack of understanding of the terminology or a reticence to label patients in this way, perhaps because it may make management more challenging.

Another possible problem with the terminology of MUS is that it suggests a commonality of diagnosis and therefore perhaps treatment. In reality, each patient has a unique presentation and will require tailored management [44]. Although complex, time taken in developing teaching around MUS has the potential to improve many consultations, resulting in better outcomes for patient and clinican as well as likely financial savings for the health care system.

## Conclusion

Despite evidence that clinicians struggle with managing patients with MUS, the current biomedical model of medical education continues to produce clinicians who rely on a biomedical model of decision making and in turn role-model this to students through the informal curriculum. This study has described the emotional impact this can have on trainees and the potential increased burden on the health care system through inappropriate referrals and sub-optimal patient care.

## Additional file

Additional file 1:
**MUS Attitudinal Questionnaire.** (DOC 1663 kb)
