# Supplementary figures and images for: “You kind of want to fix it don’t you?” Exploring general practice trainees’ experiences of managing patients with medically unexplained symptoms
Source: BMC Med Educ. 2016 Jan 25;16:27. doi: 10.1186/s12909-015-0523-y (PMC4727318; doi:10.1186/s12909-015-0523-y)

Appendix: MUS Attitudinal Questionnaire


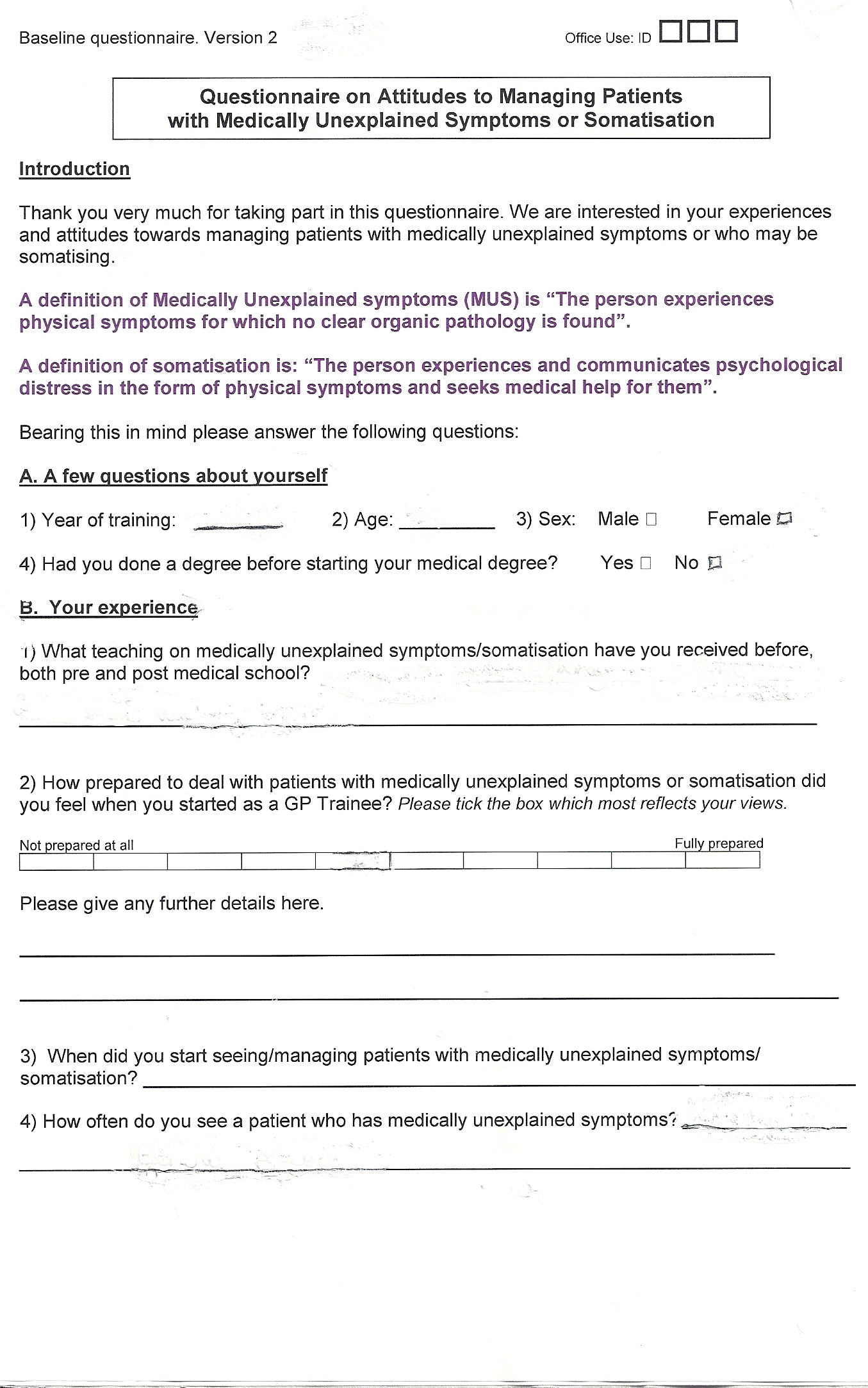


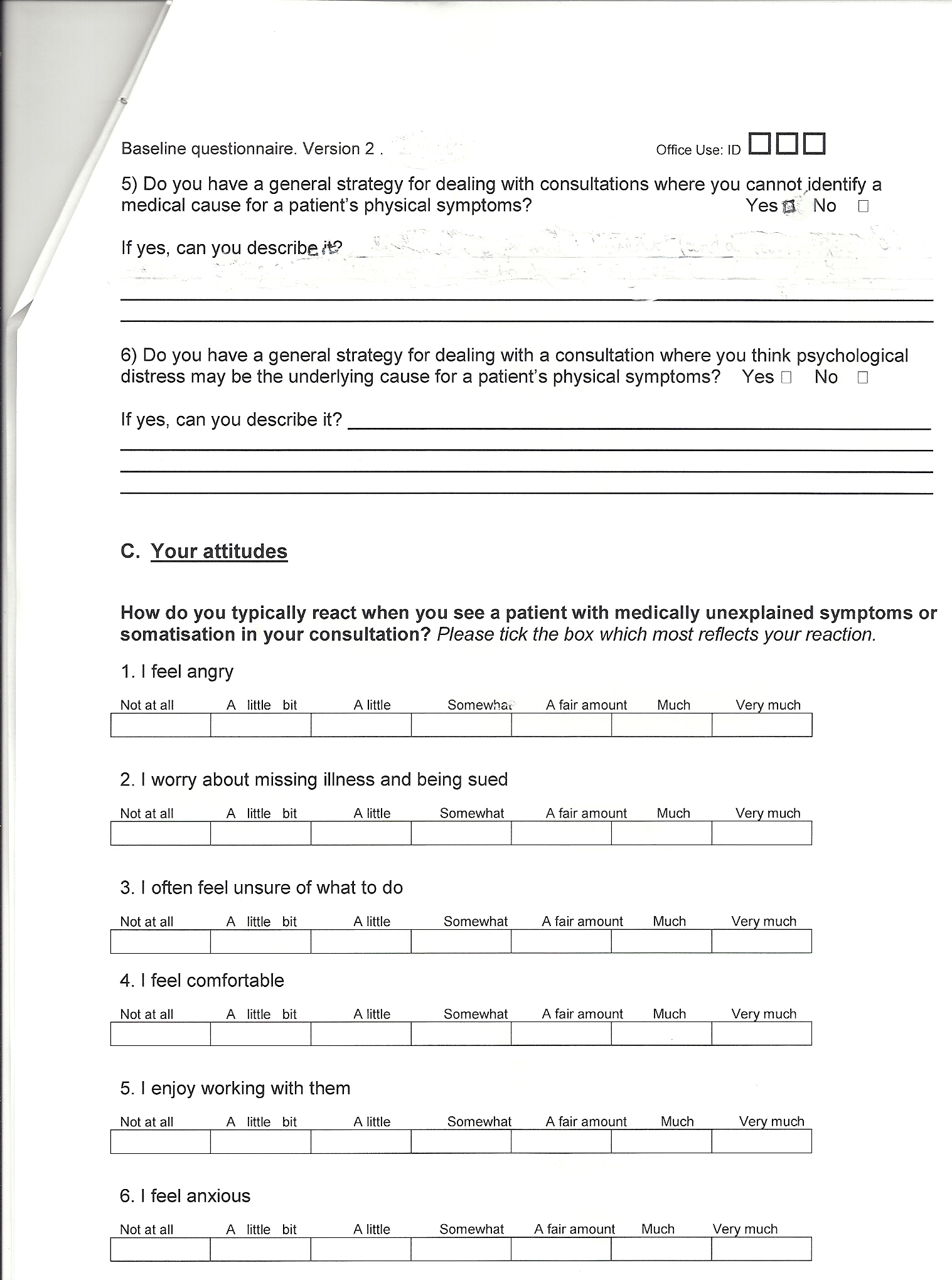


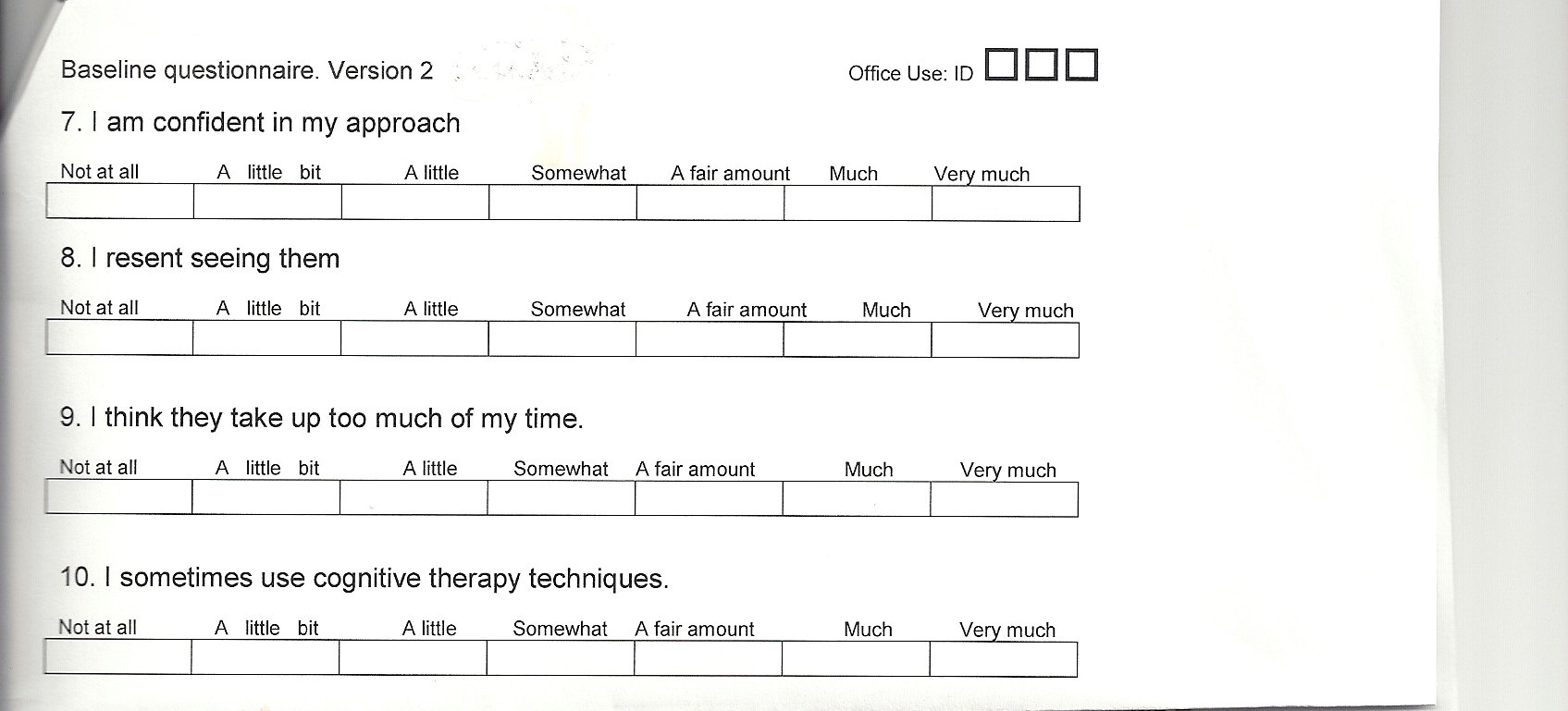

Supplement: Additional file 1: — MUS Attitudinal Questionnaire. (DOC 1663 kb) [file 12909_2015_523_MOESM1_ESM.doc]
